# Neutrophils activated by BJcuL, a C-type lectin isolated from *Bothrops jararacussu* venom, decrease the invasion potential of neuroblastoma SK-N-SH cells *in vitro*

**DOI:** 10.1590/1678-9199-JVATITD-2019-0073

**Published:** 2020-05-11

**Authors:** Jessica Ohana Lemes Carneiro-Goetten, Bruna Santos Rodrigues, Rodrigo Amauri Nogoceke, Thatyanne Gradowski do Nascimento, Andrea Novais Moreno-Amaral, Patricia Maria Stuelp-Campelo, Selene Elifio-Esposito

**Affiliations:** 1 Programa de Pós-graduação em Ciências da Saúde, Pontifícia Universidade Católica do Paraná (PUCPR), Curitiba, PR, Brazil.; 2 Escola de Ciências da Vida, Pontifícia Universidade Católica do Paraná (PUCPR), Curitiba, PR, Brazil.

**Keywords:** Snake venom, Neuroblastoma, SK-N-SH cells, C-type lectin, Polymorphonuclear leukocyte, Neoplasm invasion

## Abstract

**Background:**

Neuroblastoma is a pediatric tumor with a mortality rate of 40% in the most aggressive cases. Tumor microenvironment components as immune cells contribute to the tumor progression; thereby, the modulation of immune cells to a pro-inflammatory and antitumoral profile could potentialize the immunotherapy, a suggested approach for high-risk patients. Preview studies showed the antitumoral potential of BJcuL, a C- type lectin isolated from *Bothrops jararacussu* venom. It was able to induce immunomodulatory responses, promoting the rolling and adhesion of leukocytes and the activation of neutrophils.

**Methods:**

SK-N-SH cells were incubated with conditioned media (CM) obtained during the treatment of neutrophils with BJcuL and fMLP, a bacteria-derived peptide highly effective for activating neutrophil functions. Then we evaluated the effect of the same stimulation on the co-cultivation of neutrophils and SK-N-SH cells. Tumor cells were tested for viability, migration, and invasion potential.

**Results:**

In the viability assay, only neutrophils treated with BJcuL (24 h) and cultivated with SK-N-SH were cytotoxic. Migration of tumor cells decreased when incubated directly (p < 0.001) or indirectly (p < 0.005) with untreated neutrophils. When invasion potential was evaluated, neutrophils incubated with BJcuL reduced the total number of colonies of SK-N-SH cells following co-cultivation for 24 h (p < 0.005). Treatment with CM resulted in decreased anchorage-free survival following 24 h of treatment (p < 0.001).

**Conclusion:**

Data demonstrated that SK-N-SH cells maintain their migratory potential in the face of neutrophil modulation by BJcuL, but their invasive capacity was significantly reduced.

## Background

BJcuL is a lectin isolated from *Bothrops jararacussu* snake venom. It is a typical representative of the C-type animal lectin superfamily, having the carbohydrate recognition domain (CRD), and exhibiting specificity for β-d-galactosides [1]. Previous studies have shown that BJcuL has immunomodulatory effects. It can increase the adhesion and rolling of leukocytes in the endothelium of pre-capillary vessels [2], cause edema and increased vascular permeability when injected into mouse hind paw [3]. We have shown that BJcuL modulates macrophage differentiation towards a Th1 profile *in vitro,* with induction of phagocytosis, production of ROS [4], and secretion of pro-inflammatory cytokines, including TNF-α, IL-6, IL-8, and GM-CSF [4, 5]. BJcuL is also capable of modulating neutrophils to a pro-inflammatory phenotype, inducing the production of anion superoxide and increased phagocytic function [6].

In the tumor microenvironment (TME) cancer cells interact with extracellular matrix (ECM) proteins and non-tumor cells like fibroblasts, mesenchymal cells, and mature immune cells [7-9] that establish the overall characteristics of the tumor [10]. For example, cross talk between tumor cells and neutrophils can be a determinant in tumor progression. The first direct evidence of the cytotoxic effects of neutrophils on tumor cells was reported in 1972 [11], and since then many studies have contributed to this knowledge, as reviewed by Souto et al. [12]. Current data support a dual role for neutrophils in cancer biology, in which cytotoxic neutrophils, called N1, contribute to tumor rejection or increase immunological memory, thus combating tumor progression, and, on the other side of this spectrum, N2 neutrophils may enable tumor development, invasion, and metastasis [13]. 

Neuroblastoma (NB) is an extracranial tumor that may present during fetal development or early after birth, derived from the neural crest neuroepithelial cells [14]. It is characterized by heterogeneity and a broad spectrum of clinical behaviors ranging from spontaneous regression without any medical intervention to treatment resistant tumors with metastatic spread and poor patient survival [15-17]. Although there have been advances in the study, diagnosis, and treatment of NB, most patients with advanced disease do not enter remission even after treatment with multimodal therapies [18], which may include immunotherapy in the post-consolidation phase [19-22].

In this study, we investigate the potential of BJcuL to induce an N1 or antitumoral phenotype in neutrophils by analyzing the migration and invasion capabilities of NB cells following treatment and, thus, highlight the potential of using animal toxins and neutrophil modulation and showcase their use as effective weapons against chemotherapy-resistant solid tumors.

## Methods

### Materials

RPMI 1640 medium, fetal bovine serum (FBS), penicillin/streptomycin and trypsin were purchased from GIBCO (USA). Histopaque^®^ 1077 and 1119, fMLP (N-formyl-L-methionyl-L-leucyl-L-phenylalanine), DCFH-DA (2′,7′-Dichlorofluorescin diacetate) and methylene blue were all from Sigma-Aldrich (USA). 

### Cells

Human neuroblastoma cells SK-N-SH (ATCC^®^ HTB-11™) were cultured in RPMI 1640 medium supplemented with 100 IU/mL penicillin and 0.1 mg/mL streptomycin. Heat inactivated fetal bovine serum (FBS) was added to a final 10% concentration for culture expansion steps, or 2% for the scratch wound healing assay. The cultures were maintained in a humid incubator at 37°C, with 5% CO_2_.

**Figure 1.**
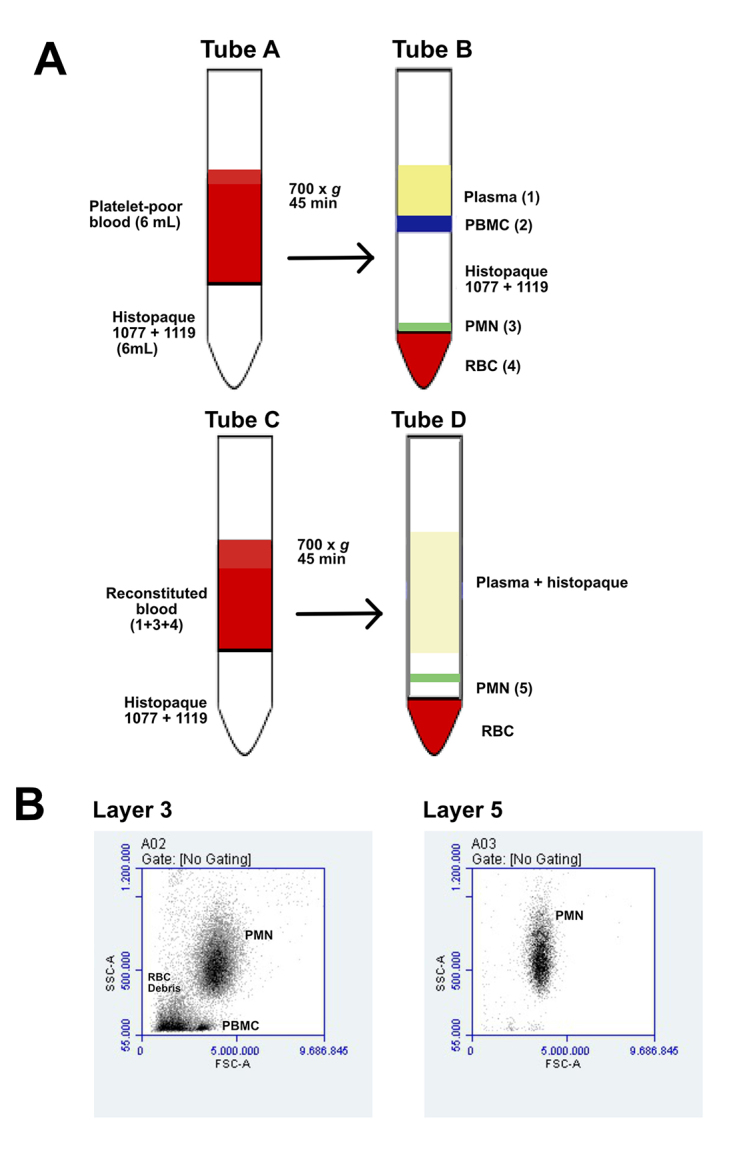
Isolation of neutrophils from human peripheral blood. Whole blood underwent a prior centrifugation step to isolate plasma and remove platelets. **(A)** Platelet-poor blood was used for the first fractionation step using Histopaque^®^ 1077 and 1119, resulting in four distinct layers: plasma, peripheral blood mononuclear cells (PBMC), polymorphonuclear neutrophils (PMN), and red blood cells (RBC). Plasma, PMN and RBC were reunited for a second isolation to allow us to obtain a PMN-enriched fraction. **(B)** Flow cytometry analysis of various fractions. Dot plot showing the mixture of PMN, RBC and PBMC in layer 3, and the high concentration of PMN (layer 5).

Polymorphonuclear neutrophils (PMN) were isolated from peripheral blood of healthy adults aged 20-40 years using a Histopaque^®^ density gradient as described by the manufacturer with minor modifications (Figure 1A). Peripheral blood was centrifuged at 1000 x *g* for 20 min, and plasma was then centrifuged at 2000 x *g* for 10 min to remove platelets. Plasma was then returned to the collection tube and homogenized with the remaining blood cells. Platelet-poor blood (6 mL) was added to tubes containing Histopaque 1119 + 1077 (6 mL), and centrifuged at 700 x *g* for 45 min. The PMN layer was collected and centrifuged at 700 x *g* for 5 min to allow for the removal of residual Histopaque^®^, and then mixed back into red blood cells (RBC) and plasma for a second round of centrifugation aiming to a better recovery of PMN without contamination with RBC or peripheral blood mononuclear cells (PBMC). 

Aliquots of the PMN populations were analyzed on an Accuri^TM^ C6 cytometer (BD Biosciences) for size (FSC, forward scatter) and complexity (SSC, side scatter) characterization (Figure 1B). A differential blood cell count (ABX MICROS 60 automatic counter) was performed before PMN isolation to verify that the cells were not pre-activated, based in the number of PMN cells in the complete blood count (CBC). Any alteration in percentage of PMN can indicate infection and thus a possible pre-activation of those cells. Therefore, only the blood which presented differential count with normal percentages of PMN was used.

### ***Bothrops jararacussu* lectin (BJcuL)**

*B. jararacussu* venom was supplied by the Butantan Institute, São Paulo, Brazil. BJcuL was purified by affinity chromatography using an agarose-D-galactose column and characterized as previously described by Elifio-Esposito et al*.* [2].

### Stimulation of SK-N-SH cells

SK-N-SK cells (1 x 10^6^ cells/mL) were co-cultured with neutrophils (2.5 x 10^5^ cells/mL) on a 24-well plate (300 µL/well), for 24 h in RPMI with or without BJcuL (2.5 μg/mL) or fMLP (10 µM).

To obtain the conditioned media (CM), neutrophils (2.5 x 10^5^ cells/mL) cultured in a 6-well plate (1 mL/well) were incubated with BJcuL (2.5 μg/mL), fMLP (10 µg/mL), or 2% RPMI (untreated control) for 1 h or 24 h. The supernatants were collected, centrifuged (2000 x *g* for 5 min), filtered (0.22 µm), and kept frozen at -80°C until use. After the removal of the CM, neutrophils from each treatment were stained with a DCFH-DA probe, according to the manufacturer's instructions and analyzed using an Accuri flow cytometer (BD Biosciences), to estimate the intracellular hydrogen peroxide level. For treatment of NB cells, CM was diluted 1:2 (v/v) with RPMI (2% FBS) and added to wells containing the SK-N-SH tumor cells and incubated for 24 h.

### Scratch Wound Healing (SWH)

*In vitro* cell migration was evaluated using the scratch wound healing (SWH) method, according to the previously described protocol [23], with the following modifications. NB cells (3.5 x10^5^ cells) were transferred to a 24-well plate, and after 24 h the cell monolayer was mechanically scratched. Detached cells were removed by washing with RPMI. Cells were then treated for 24 h, as described before. After treatment, cells were washed with RPMI and maintained in culture for another 24 h. Photomicrographs of the wounds were taken immediately after their formation (0 h) and at 48 h after (T48) using an EVOS^®^ XL microscope. The wound width at T0 and T48 was measured using the ImageJ software plugin (NIH, USA), and the migration distance was determined as the difference in width between T0 and T48.

### Cell viability assay

 SK-N-SH cell viability was determined directly on the plate using the SWH test, following the protocol described previously [24], with some modifications. Briefly, after completion of the SWH assay, medium was removed, and 200 µL of methanol was added, and allowed to fix for 10 min. Cells were then stained with 300 µL of 0.05% methylene blue for 10 min. Plates were washed by immersion and remained at room temperature for drying for 24 h. About 300 µL of 0.1 M HCl was added and plates were agitated for 10 min. Following which 100 µL from each well was transferred to a 96-well plate for absorbance reading on a microplate reader (ThermoPlate, TP Reader), at a wavelength of 630 nm.

### Soft agar anchorage-free survival test

The soft agar test was performed in 48-well plates as previously described [25]. The bottom layer was made up of 250 µL of 2X RPMI medium containing 20% of FBS and 250 µL of 1.2% agar in water. The upper layer consisted of 250 µL of 2X RPMI medium (20% FBS), 2 x 10^3^ SK-N-SH cells, 125 μL of 1.2% agar and 125 μL of water. Every 72 h, 30 µL of 1X RPMI medium (10% FBS) was added to form the feeding layer. SK-N-SH cells were treated by the direct (BJcul or fMLP) or indirect method (CM) for 24 h prior to being added to the upper layer. Results were obtained by direct colony counting following 14 days of culture.

### Statistical Analysis

The results for the quantitative tests are represented by the mean ± standard deviation. Statistical analysis was performed by ANOVA, followed by multiple comparison testing using the Tukey's or Dunnett’s test. Statistical significance was assigned for values where p < 0.05. Analyses were performed using GraphPad Prism Software, version 8.0 (La Jolla California, USA).

## Results

### SK-N-SH cell migration is reduced in the presence of neutrophils and BJcuL reverses this effect

The effects of BJcuL on NB cell migration were assessed by SWH assays following two distinct stimulation procedures. In the indirect method, NB cells were incubated for 24 h with conditioned media (CM) generated by culturing neutrophils in RPMI medium containing BJcuL or fMLP for 1 h (NE1h-CM) or 24 h (NE24h-CM). In the direct method, NB cells were co-cultivated with neutrophils for 24 h, in the presence of BJcuL or fMLP. We also verified the direct effects of BJcuL or fMLP on tumor cells, in the absence of neutrophils. 

**Figure 2.**
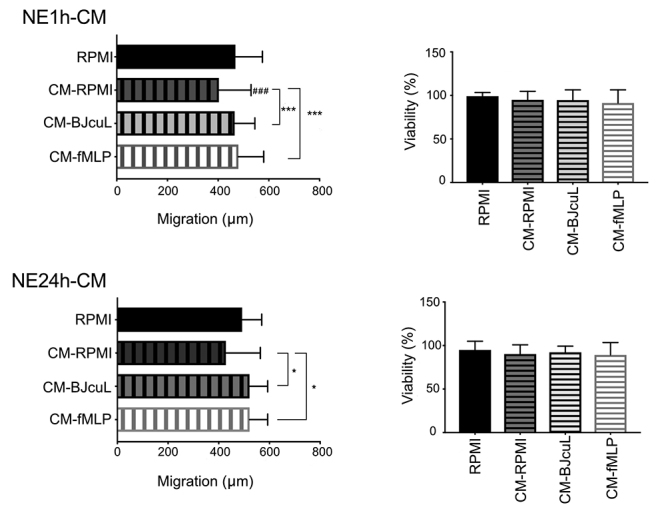
Analysis of migration and viability of SK-N-SH incubated with neutrophil conditioned media (CM). NB cells (1x10^6^ cell/mL) were incubated for 24 h in a 24-well plate (300 µL/well) with 1:2 (v/v) neutrophil-conditioned media (CM). CMs were generated by culturing neutrophils in RPMI, 2.5 µg/mL BJcuL or 10 µM fMLP for 1 h (NE1h-CM) or 24 h (NE24h-CM). Quantitative results are represented as means ± standard deviation (SD) of the migration distance (µm), determined as the difference in wound width at T0 and T48 (T0-T48; n = 12). After the completion of the migration assay, the viability assay of the NB cells in each condition was established. Viability results are represented as mean ± SD (n = 10) of viable cells normalized to the untreated control (RPMI). Data were obtained from at least three independent assays. Statistical analysis was performed by one-way ANOVA and Tukey's test with significance reported as *p < 0.05; **p < 0.005 and ***p < 0.001. # indicates difference comparing with RPMI control.

For the indirect assay, media obtained from non-stimulated neutrophils reduced the migration rate of SK-N-SH cells when compared to RPMI control, although a significant difference was found only for the 1h group. Even so, for both 1 h or 24 h conditions, CM from neutrophils treated with BJcuL and fMLP improved cell migration compared to CM-RPMI, restoring the migration capacity, closer to RPMI control (Figure 2 and Additional file 1). Cell viability analysis was done to exclude the possibility that the behavior observed in the SWH assay was the result of cellular proliferation. The viability of the neuroblastoma cells was not affected in treatments with conditioned media. 

**Figure 3.**
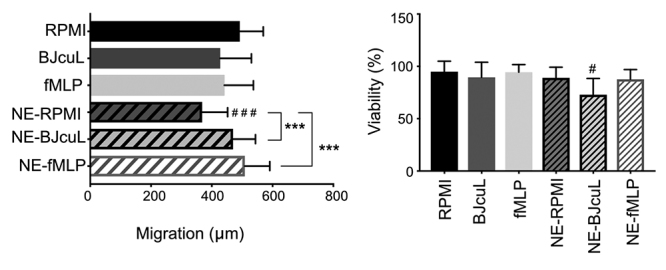
Analysis of migration and viability of SK-N-SH co-cultured with neutrophils. SK-N-SH cells (1 x 10^6^ cells/mL) were incubated with RPMI, 2.5 µg/mL BJcuL or 10 µM fMLP alone or in co-culture with human neutrophils (NE; 2.5 x 10^5^ cells/mL) for 24 h in a 24-well plate (300 µL/well). Results represent mean ± SD of the migration distance, determined as T0-T48 (n = 12). Viability results are represented as mean ± SD (n = 10) of viable cells normalized to the untreated control (RPMI). Data were obtained from at least three independent assays. Statistical analysis was performed by one-way ANOVA and Tukey's test with significance shown as *p < 0.05 and ***p < 0.001. # indicates difference comparing with RPMI control.

Following the same pattern, the direct incubation with neutrophils (NE-RPMI) reduced SK-N-SH migration, which was restored to RPMI control values when BJcuL or fMLP were added to the system (Figure 3). When BJcuL was used as a direct stimulant for 1 h (data not shown) or 24 h, it did not change the migration rate of SK-N-SH cells, when compared with untreated cells (RPMI). NB cell viability was not affected by BJcuL alone, but when it was added to the neutrophil co-culture, we observed an almost 20% reduction in the number of viable cells (Figure 3).

### Neutrophils treated with BJcuL reduce the invasion of SK-N-SH cells

**Figure 4.**
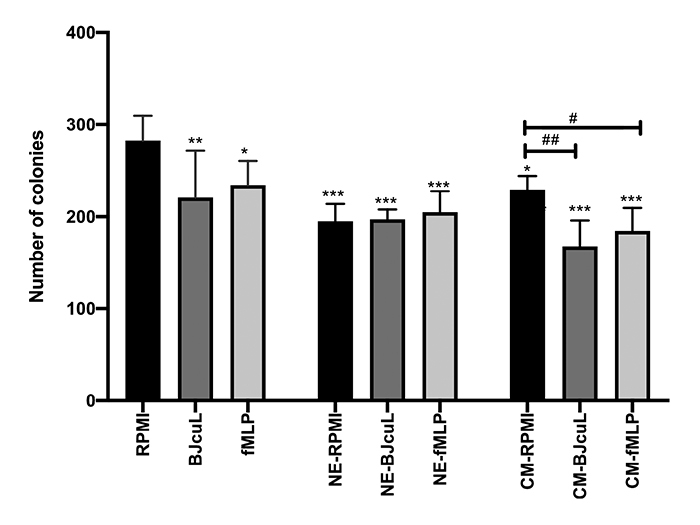
Anchorage-free survival assay of SK-N-SH cells after incubation with various stimuli. SK-N-SH cells (2 x 10^3^ cells/well) were incubated for 24 h with RPMI, BJcuL (2.5 μg/mL) or fMLP (10 μM), or conditioned neutrophil media (CM-RPMI, CM-BJcuL or CM-fMLP). SK-N-SH cells were also stimulated for 24 h with neutrophils in association with BJcuL, fMLP or 2% RPMI. After treatment SK-N-SH cells were dissociated, washed and plated on a mirror plate containing soft agar layers. Results are represented as mean ± SD (n = 8) of the total number of colonies counted in each well. Data were collected from at least two independent assays. The statistical comparison among groups was performed by one-way ANOVA (p < 0.05). Dunnetts's test was used to compare all treatments with the RPMI group. Significance is represented as *p < 0.05; **p < 0.005 and ***p < 0.001. # represents the comparison of treatments with the respective control (CM-RPMI).

The invasion capacity of tumor cells *in vitro* was determined using the soft agar method. The principle of this method is that malignantly transformed cells lose their anchorage dependence and can grow and form colonies within a semi-solid agar matrix. All tested conditions, i.e., with direct or indirect stimulation of SK-N-SH cells, led to a decrease in the number of colonies when compared to the RPMI group. After 24 h of co-culture with neutrophils in RPMI alone (NE-RPMI) or in the presence of BJcuL or fMLP (NE-BJcuL; NE-fMLP), the invasion capacity of SK-N-SH was reduced by approximately 30%, when compared to the untreated control (RPMI). Incubation of tumor cells with the conditioned media (CM-RPMI) also reduced the number of colonies, which was more pronounced for the CM from neutrophils treated with BJcuL (CM-BJcuL) and fMLP (CM-fMLP), with a drop of 40% and 35% in the number of colonies, respectively, when compared with the RPMI control (Figure 4). 

### BJcuL stimulates the production of hydrogen peroxide by neutrophils

**Figure 5.**
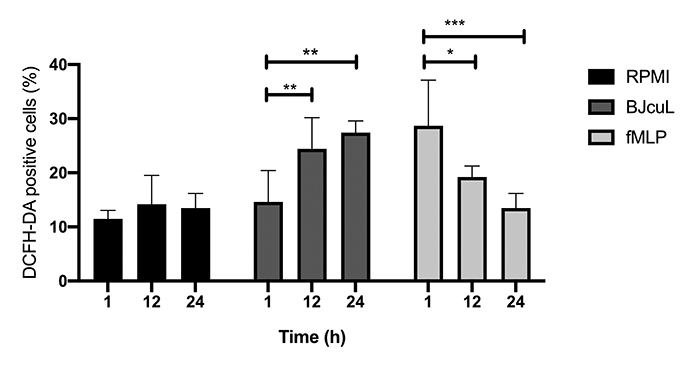
Determination of neutrophil intracellular hydrogen peroxide production. Neutrophils were treated with BJcuL (2.5 µg/mL) or fMLP (10 µM) for 1, 12, and 24 h and stained with DCFH-DA for flow cytometric analysis. Results are represented as mean ± SD. Statistical analysis was done using ANOVA and Dunnett’s test for comparison of treated groups with untreated control (RPMI). Statistical significance is represented as *p < 0.05, and ***p < 0.001.

Neutrophils employed for the production of the CMs had their pro-inflammatory activity assessed by monitoring ROS production after incubation with BJcuL and fMLP for 1h and 24 h. It was added a 12h-stimulation condition to uncover the profile of ROS production along time, even if no CM was generated for this time point. BJcuL and fMLP stimulated cells showed a higher level of intracellular hydrogen peroxide when compared to the untreated control (Figure 5). For fMLP treated cells there was a significant increase after 1 h of stimulation, which gradually reduced to the 24 h time point. BJcuL produced the opposite profile, with low intracellular ROS content after 1 h of stimulation, and a statistically significant increase in ROS production after 12 h and 24 h.

## Discussion

C-type lectins are among the main components exhibiting pro-inflammatory activity in viper snake venoms [1, 26-28]. The pro-inflammatory potential of *B. jararacussu* venom lectin was reported previously [2-5, 29], which includes the induction of neutrophil polarization [6]. In this study, we demonstrated that the modulation of neutrophils by BJcuL reduced the viability and invasion capacity of human neuroblastoma cells *in vitro*. As neutrophil polarization is likely driven by TME [30, 31], neutrophils were tested in the presence and absence of tumor cells.

Exogenous chemotaxins, as fMLP , attract neutrophils to inflammatory sites to recognize and kill pathogens through a combination of cytotoxic mechanisms [32, 33]. Emerging evidence supports that neutrophils may also be cytotoxic to tumor cells [30, 34]. Our results show that a significant reduction in SK-N-SH cell viability occurs when they are cultivated directly with neutrophils for 24 h in the presence of BJcuL, but not fMLP (Figures 2 and 3), which is consistent with the increasing levels of intracellular hydrogen peroxide found by BJcuL-treated neutrophils within 24 h (Figure 5). ROS-mediated cell killing was shown to be dependent on tumor cell expression of TRPM2, an H2O2-dependent calcium channel, which upon activation results in a lethal influx of calcium ions into the cell [35, 36]. The conditioned media generated by the cultivation of neutrophils did not affect NB cell viability, which may be due to the different in the concentration of the cytotoxic agents, as the CM was diluted for the incubation with NB cells.

As previously reported, BJcuL reduces the degree of late apoptosis in neutrophils, decreasing in 50% in the number of apoptotic cells after 24 h of incubation [6]. A recent study showed that mediators of delayed apoptotic cell death pathways of N1 cells can keep the system in the safe-guard immune N1 zone [37]. BJcuL could induce PMNs to exert a direct cytotoxic effect on tumor cells, and also favor the maintenance of an activated profile with effects on other aspects of tumor progression.

Metastasis remains the leading cause of death for patients with cancer [38]. It is a multistep process that involves the collective cell migration, which heavily relies on the cooperation of the cytoskeleton, the cellular surface adhesion proteins, and the extracellular matrix (ECM) components [39, 40]. The secretion of cytokines by neutrophils, like components of the IL-6 family and IL-8, for example, as well as growth factors [41-43], can promote ECM remodeling in the TME [94]. Furthermore, mature neutrophils possess granules containing a reservoir of enzymes, including myeloperoxidase (MPO), neutrophil elastase (NE), and also metalloproteinases (MMPs), capable of remodeling the ECM by stabilizing integrins [44]. Cell adhesion mediated mainly through the interaction of integrin receptors with their ECM ligands is a requirement for many cell types to proliferate and survive [45].

In this study, we found by SWH assay, a significant reduction in tumor cell migration when SK-N-SH cells were incubated directly with untreated neutrophils. These results were unexpected considering that circulating "normal" PMNs are described not to affect the adhesion and migration of tumor cells [46]. Regardless, the addition of either BJcuL or fMLP, both described to increase the pro-inflammatory potential of neutrophil, increased the migration capacity of NB cells. Neutrophil-tumor cell interaction was not the determining factor in this case, as the direct and indirect stimulation resulted in the same way. Furthermore, anchorage-free survival assays showed that incubation with untreated or stimulated neutrophils reduced the invasive power of neuroblastoma cells, which is even greater in the presence of BJcuL or fMLP. 

The recognition of specific glycans by lectins represents a key event in a variety of biological phenomena involving cell-cell and cell-ECM component interactions [47, 48]. BJcuL, as Galatrox, another C-type lectin derived from *B. atrox* snake venom, can interact with glycans on neutrophils and macrophages surfaces, as well as with ECM proteins. They have been described also as stimulants of the neutrophils release of pro-inflammatory mediators, others than ROS, such as IL-6, and TNF-α [1, 2, 6, 49]. These properties together could favor and stabilize cellular adhesion, facilitating migration but also reducing anchorage-free survival.

The complexity of the tumor microenvironment cannot be easily recapitulated *in vitro*. The master signals exchanged between various cell types within the tumor mass is critical, and evidence supports that cytokines control pro-tumor versus anti-tumor immune responses by polarizing neutrophil subpopulations to N1 or N2 phenotypes. However, even with the limitations, we reinforce in this study, that BJcuL affects the behavior of neutrophils *in vitro* and that such action can be a mechanism to be explored in order to better understand the processes involved in neutrophils anti-invasive action.

## Conclusion

Our results showed that BJcuL-treated neutrophils could significantly affect the behavior of neuroblastoma cells *in vitro*. Viability was reduced when neutrophils were co-cultivated with the tumor cells, while the increment of cellular migration and the reduction of anchorage-free survival were also observed by the incubation of tumor cells with the neutrophil-conditioned media. Efforts must be made to identify the signaling molecules exposed on the cellular surface or secreted by neutrophils in order to explain the presented data.

### Abbreviations

CBC: complete blood count; CM: conditioned media; CRD: carbohydrate recognition domain; ECM: extracellular matrix; FBS: fetal bovine serum; FSC: forward scatter; MMPs: metalloproteinases; MPO: myeloperoxidase; NB: neuroblastoma; NE: neutrophil elastase; PBMC: peripheral blood mononuclear cells; PMN: polymorphonuclear neutrophils; RBC: red blood cells; SSC: side scatter; SWH: scratch wound healing; TME: tumor microenvironment.

## Supplementary material

The following online material is available for this article:

Additional file 1. Representative photomicrographs of the wounds to the SK-N-SH cells monolayer taken at T0 and T48.
