# Supplementary material for: Neutrophils activated by BJcuL, a C-type lectin isolated from Bothrops jararacussu venom, decrease the invasion potential of neuroblastoma SK-N-SH cells in vitro
Source: J Venom Anim Toxins Incl Trop Dis. 2020 May 11;26:e20190073. doi: 10.1590/1678-9199-JVATITD-2019-0073 (PMC7216824; doi:10.1590/1678-9199-JVATITD-2019-0073)
Supplement: Additional file 1. [file 1678-9199-jvatitd-26-e20190073-s1.pdf]

Supplementary Material to "Neutrophils activated by BJcuL, a C-type lectin isolated from *Bothrops jararacussu* venom, decrease the invasion potential of neuroblastoma SK-N-SH cells *in vitro*"

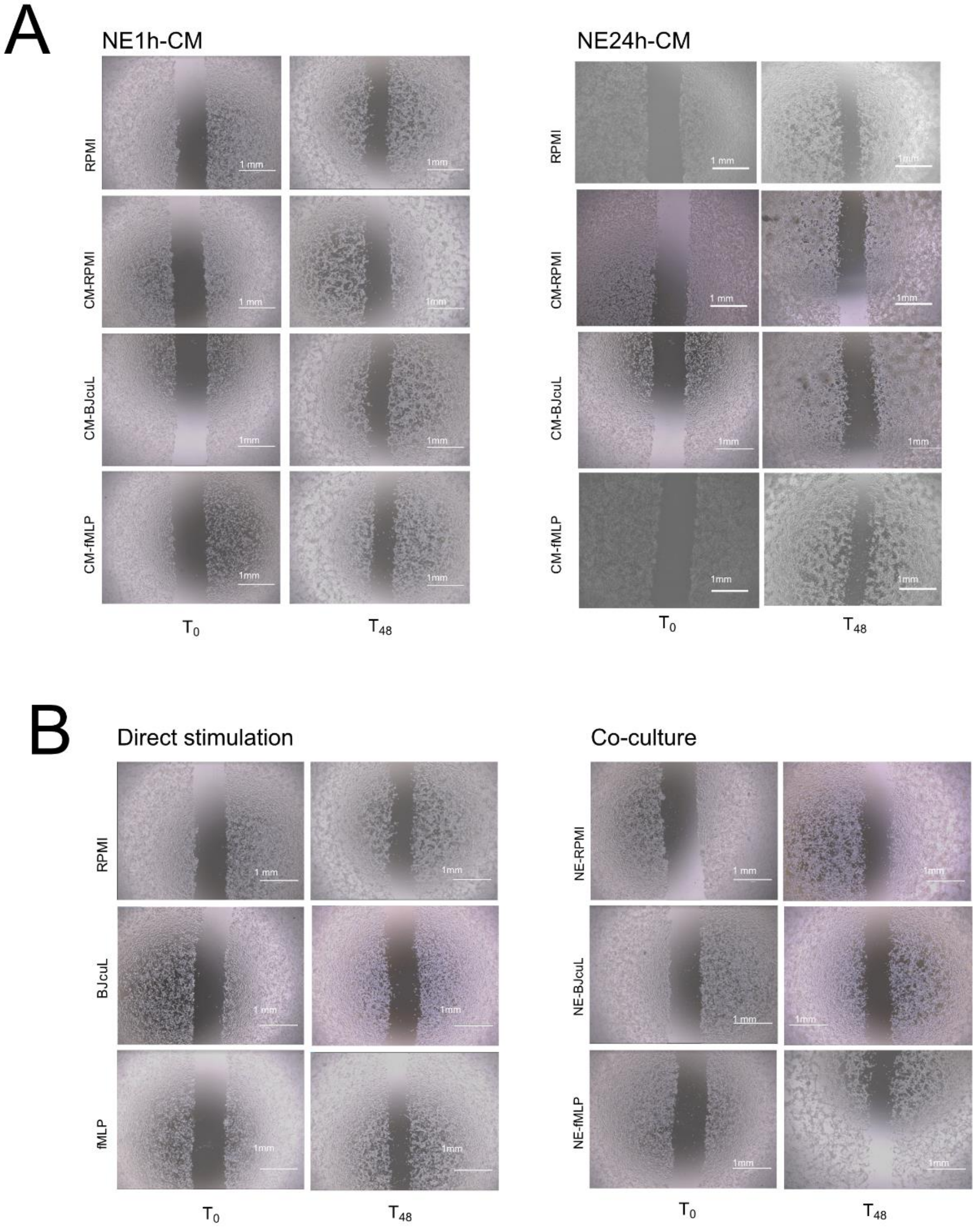

Additional file 1. Representative photomicrographs of the wounds to the SK-N-SH cells monolayer taken at T0 and T48. **(A)** NB cells (1x10^6 cell/mL) were incubated for 24 h in a 24-well plate (300 µL/well) with 1:2 (v/v) neutrophils-conditioned media (CM). CMs were generated by culturing neutrophils in RPMI, 2.5 µg/mL BJcuL or 10 µM fMLP for 1 h (NE1h-CM) or 24 h (NE24h-CM). **(B)** SK-N-SK cells were incubated with RPMI, 2.5 µg/mL BJcuL or 10 µM fMLP alone or in co-culture with human neutrophils (NE; 2.5 x 10^5 cells/mL) for 24 h in a 24-well plate (300 µL/well).
